# Mobile Carrier Structure for Facilitated CO_2_ Transport in Mixed Ionic Liquid Membrane Composed of 1‐Ethyl‐3‐Methylimidazolium Acetate and Diamine‐Functionalized Ionic Liquids

**DOI:** 10.1002/cssc.70685

**Published:** 2026-05-15

**Authors:** Yu Nagai Kanasaki, Yuki Kohno, Takashi Makino

**Affiliations:** ^1^ Research Institute for Chemical Process Technology National Institute of Advanced Industrial Science and Technology (AIST) 4‐2‐1, Nigatake, Miyagino‐ku Sendai Miyagi Japan

**Keywords:** direct air capture, facilitated transport, gas separation, ionic liquids, membranes

## Abstract

Achieving global carbon neutrality by 2050 requires technologies capable of capturing CO_2_ at atmospheric concentrations. Membrane‐based direct air capture (DAC) offers an energy‐efficient route, and facilitated transport membranes (FTMs) using ionic liquids (ILs) are promising owing to their structural diversity with tunable reactivity. This study examines the influence of diamine‐functionalized IL carrier structure and process conditions on CO_2_ separation in DAC‐relevant environments. FTMs are prepared by impregnating a porous polymer support with blended ILs composed of 1‐ethyl‐3‐methylimidazolium acetate ([C_2_mim][AcO]) and various diamine‐functionalized ILs. The relationship between CO_2_ solubility and CO_2_ permeability of the FTMs shows an optimum solubility range that affords high CO_2_ permeability. When CO_2_ solubility is excessive, CO_2_ permeability becomes relatively low. Suppressing the solubility through molecular modification by introducing hydroxyethyl groups or adjusting the diamine spacer effectively shifts the solubility value into a suitable range that enables higher CO_2_ permeability. Further decrease in the solubility results in low permeability. The effects of both temperature and humidity on the separation performance are also assessed, and optimal temperature conditions and mixing ratios are identified for superior CO_2_ permeability. These findings clarify the chemical structure–performance relationships and inform the design of efficient FTMs for DAC applications.

## Graphical abstract

Mixed ionic liquid membranes combining 1 ethyl‐3‐methylimidazolium acetate with diamine‐functionalized ionic liquids enable facilitated CO_2_ transport for membrane based direct air capture. Tuning diamine terminal groups and spacer length establishes an optimal range in CO_2_ solubility that enhances CO_2_ permeability, and one hydroxyethyl terminal group with an ethylene spacer maximizes the separation performance.

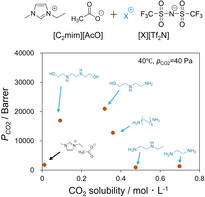

## 1 Introduction

Achieving global carbon neutrality by 2050 will require not only energy savings and large‐scale deployment of renewable energy but also technologies for capturing CO_2_. According to the International Energy Agency’s Net Zero by 2050 scenario, global CO_2_ capture is projected to increase from ∼40 Mt in 2020 to ∼1.2 Gt by 2030 and ∼6.3 Gt by 2050 [1]. Target CO_2_ concentrations range from atmospheric levels (∼0.04%) to industrial streams (up to ∼50%), motivating the development of separation platforms that operate effectively across widely varying conditions. Direct Air Capture (DAC) is a promising CO_2_ capture technology owing to its flexibility and ability to remove CO_2_ directly from the air. Two leading DAC technologies, absorbents and adsorbents, are mainly investigated for large‐scale deployment. On the other hand, several emerging DAC technologies are in the early stages of development. A membrane‐based DAC (m‐DAC) has attracted growing attention because membrane separation can be driven primarily by electrical energy, without the need for direct thermal energy input, and thus provides an energy‐efficient route in terms of process configuration and sustainable operating conditions (e.g., when powered by renewable electricity to operate air‐moving fans and vacuum pumps for generating a pressure difference) [2, 3, 4]. The m‐DAC systems with high separation performance are particularly promising for CO_2_ capture and separation in small‐scale, distributed applications [5, 6, 7].

Facilitated transport membranes (FTMs) have emerged as promising membranes for CO_2_ separation, especially at low CO_2_ partial pressures. By incorporating reactive carriers that selectively bind and shuttle CO_2_, FTMs simultaneously realize high CO_2_ permeability and high CO_2_/N_2_ selectivity.

Two archetypes are commonly distinguished: mobile‐carrier systems, in which carrier molecules migrate within the permeating phase, and fixed‐carrier systems, in which carriers are immobilized in a solid or gel matrix. Fixed‐carrier FTMs, typically based on polymer‐bound amines or ionizable functional groups, have been widely studied and applied to post‐combustion CO_2_ separation, where mechanical robustness and process stability are advantageous [8, 9, 10]. In contrast, mobile‐carrier FTMs employ low‐molecular‐weight carriers such as amines or ionic liquids, which can migrate within the membrane phase, enabling faster reaction–diffusion cycles and higher CO_2_ transport rates at low CO_2_ partial pressures. Owing to these characteristics, mobile‐carrier FTMs are particularly attractive for DAC‐relevant separations [6, 7, 8, 9, 10, 11, 12].

Among mobile‐carrier materials, ionic liquids (ILs) have been extensively studied as task‐specific carriers to provide FTMs [6, 13, 14, 15, 16, 17]. ILs are salts that are in the liquid state near or below room temperature and are characterized by negligible vapor pressure, high thermal stability, and intrinsic ionic conductivity. By tailoring cation/anion structures and introducing reactive functional groups, ILs can chemically and reversibly bind CO_2_ while remaining non‐volatile under operating conditions.

Recent studies have demonstrated that exceptionally high CO_2_ separation performance can be achieved with IL‐based mobile‐carrier FTMs even under near‐atmospheric CO_2_ concentrations. For example, a mobile‐carrier IL membrane operated at 40% relative humidity exhibited a CO_2_ permeability of 49,100 Barrer together with a CO_2_/N_2_ selectivity of 13,200 [18], highlighting the potential of IL‐based FTMs for DAC‐relevant conditions. At the same time, the results indicate that separation performance is highly sensitive to carrier chemistry and operating environment.

Most studies on IL‐based FTMs have focused on single IL systems, exploring variations in IL chemistry, carrier functionality, and membrane morphology, thereby establishing a broad design space for CO_2_‐selective transport [7, 18, 19, 20]. We previously showed that blending a diamine‐functionalized IL bearing an *N*‐(2‐hydroxyethyl)ethylenediamine‐type carrier with 1‐ethyl‐3‐methylimidazolium acetate ([C_2_mim][AcO]) markedly enhanced separation relative to the single‐component system [21]. Under DAC‐relevant low CO_2_ partial pressures, an FTM with the binary IL achieved a CO_2_ permeability of 25,983 Barrer with a CO_2_/N_2_ selectivity of 10,059. We also investigated the CO_2_ absorption performance and underlying mechanism of the diamine‐functionalized ILs blended with [C_2_mim][AcO], and found that mixing ILs finely tunes the chemical reactivity by promoting proton‐sharing interactions, which suppress the formation of fully protonated amines and thereby increases the number of amine sites available to react with CO_2_. As a result, the mixture exhibits enhanced CO_2_ solubility and reduced heat of absorption [22].

Despite these advances, two key questions remain to be addressed for the rational design of FTMs based on the mixed ILs. The first issue is to elucidate the influence of the chemical structure of diamine carriers on the facilitated CO_2_ transport when diamine‐functionalized ILs are employed as mobile carriers. The second issue is to understand the effects of temperature and humidity, which are crucial operating conditions for controlling stable membrane performance.

**FIGURE 1:**
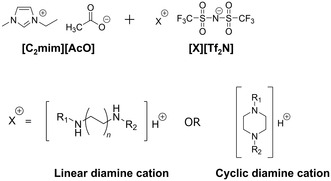
Chemical structures of the ILs used in this study.

In this study, we investigated FTMs composed of various diamine‐functionalized ILs blended with [C_2_mim][AcO]. The effects of the diamine carrier structure, specifically the terminal functional groups, main framework (linear or cyclic diamine cation), and spacer length between the amino groups, on the CO_2_ separation performance were systematically studied (Figure 1). Furthermore, the effects of temperature and humidity on CO_2_ permeability and CO_2_/N_2_ selectivity were examined. Through these investigations, this work aims to clarify the structure–performance relationships of the FTMs based on diamine‐functionalized IL mixtures, providing insights into the control of CO_2_ separation performance at low CO_2_ partial pressure.

## 2 Results and Discussion

### 2.1 Effect of Diamine Structure on CO_2_ Separation

Table 1 shows the structures and abbreviations of the diamine‐functionalized ILs used in this study. The linear diamine ILs (IL1–IL13) have spacer lengths (n) ranging from 0 to 6, and the terminal groups (R_1_ and R_2_) are hydrogen (H), ethyl group (CH_2_CH_3_), or hydroxyethyl group (CH_2_CH_2_OH). The cyclic diamine ILs (IL14–IL16) are based on a piperazine skeleton, with terminal groups (R_1_ and R_2_) consisting of either H or CH_2_CH_2_OH. Each diamine‐functionalized IL was mixed with [C_2_mim][AcO] at a molar fraction of 90:10 (10 mol% diamine‐functionalized IL) to prepare and evaluate the FTMs [21]. Hereafter, the FTMs prepared from [C_2_mim][AcO] mixed with each diamine‐functionalized IL at the specified molar fraction are referred to as “IL[number]‐[diamine IL molar fraction]” (e.g., “IL5‐10” for [C_2_mim][AcO]: IL5 with molar fraction of 90:10) for simplicity.

**TABLE 1:** Terminal structures and abbreviations of the ILs used in this study.

**Diamine IL ([X][Tf**_**2**_ **N]) abbreviation**	Cation structure	*n*	R_1_	R_2_
IL1	Linear diamine cation	0	─H	─CH_2_CH_2_OH
IL2		1	─H	─H
IL3		1	─H	─CH_2_CH_3_
IL4		1	─CH_2_CH_3_	─CH_2_CH_3_
IL5		1	─H	─CH_2_CH_2_OH
IL6		1	─CH_2_CH_2_OH	─CH_2_CH_2_OH
IL7		2	─H	─H
IL8		2	─H	─CH_2_CH_2_OH
IL9		3	─H	─H
IL10		3	─H	─CH_2_CH_2_OH
IL11		4	─H	─H
IL12		5	─H	─H
IL13		6	─H	─H
IL14	Cyclic diamine cation	—	─H	─H
IL15		—	─H	─CH_2_CH_2_OH
IL16		—	─CH_2_CH_2_OH	─CH_2_CH_2_OH

**FIGURE 2:**
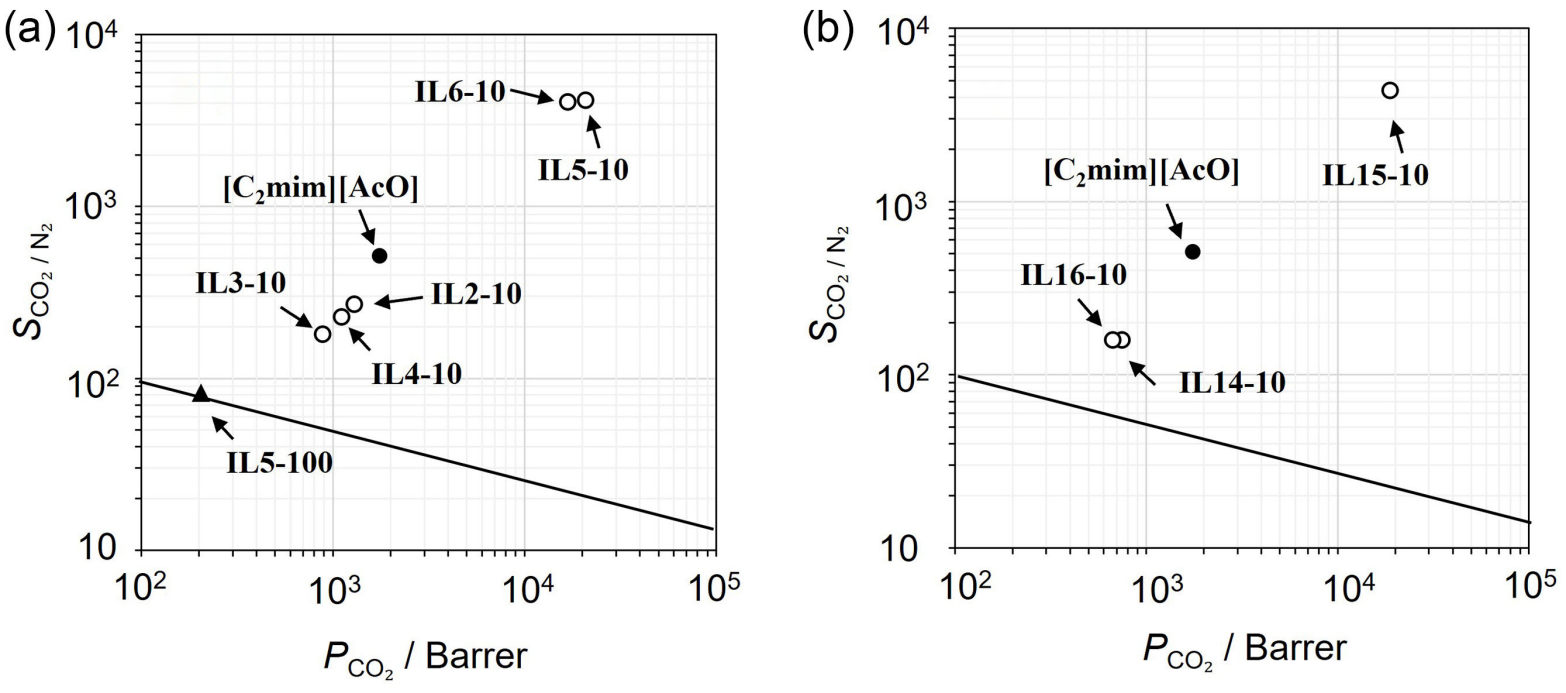
Robeson plots of the FTMs with [C_2_mim][AcO] + diamine IL mixtures (90:10 mol%): (a) mixtures of linear diamine IL(R_1_, R_2_) : IL2(H, H), IL3(H, C_2_H_5_), IL4(C_2_H_5_, C_2_H_5_), IL5(H, C_2_H_4_OH), IL6(C_2_H_4_OH, C_2_H_4_OH), where all linear diamine ILs have *n* = 1, (b) mixtures of cyclic diamine IL(R_1_, R_2_): IL14(H, H), IL15(H, C_2_H_4_OH), IL16(C_2_H_4_OH, C_2_H_4_OH). All measurements were performed at 313 K under dry conditions (water vapor pressure of <0.03 kPa) with a feed gas of CO_2_ 40 Pa in N_2_. The solid line represents the polymer upper boundary reported in Ref. [23]. Closed circles indicate the [C_2_mim][AcO] single system, and a closed triangle indicates the pure diamine IL system.

Figure 2a plots the CO_2_ permeability (*P*_CO2_) and CO_2_/N_2_ selectivity (*S*_CO2/N2_) of the FTMs containing IL mixtures with linear diamine ILs (IL2‐IL6) under the dry condition (water vapor pressure of <0.03 kPa) at 313 K, presented as Robeson plots [23]. The values of *P*_CO2_ and *S*_CO2/N2_ were shown in Table S1. All FTMs exhibited CO_2_ separation performance above the upper boundary of conventional polymer membranes [23]. The *P*_CO2_ and *S*_CO2/N2_ values decreased in the following order: IL5−10 > IL6−10 *>* [C_2_mim][AcO] > IL2−10 > IL4−10 > IL3−10. For comparison, the performance of the pure diamine IL was also evaluated using IL5 (IL5−100). Both the *P*_CO2_ and *S*_CO2/N2_ of IL5−100 were lower than those of [C_2_mim][AcO] and the IL5‐10 mixed system. Figure 2b presents the results for FTMs containing cyclic diamine ILs (IL14‐IL16) under the same conditions. IL15−10 outperformed [C_2_mim][AcO], whereas IL14−10 and IL16−10 showed inferior performance. These findings indicate that the terminal group structure on diamine carriers strongly influences the CO_2_ separation performance. Ethyl functionalization on linear diamine ILs reduced both *P*_CO2_ and *S*_CO2/N2_ compared to [C_2_mim][AcO]. In contrast, introducing one hydroxyethyl group enhanced both permeability and selectivity for both linear‐ and cyclic‐diamine systems. Two hydroxyethyl groups had contrasting effects: improvement for linear diamine systems but decline for cyclic systems. Overall, linear diamine systems tend to exhibit higher CO_2_ separation performance than cyclic ones in this study.

**FIGURE 3:**
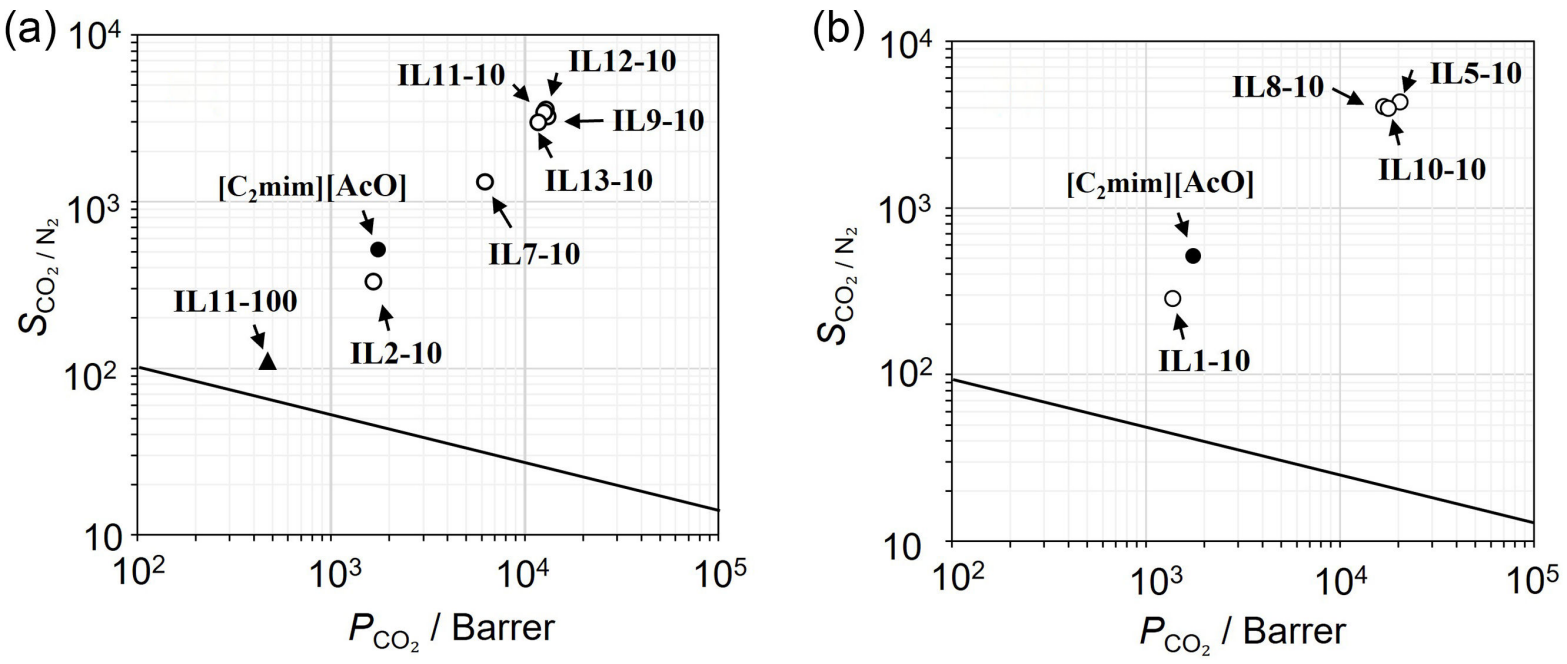
Robeson plots of the FTMs with [C_2_mim][AcO] + diamine IL mixtures (90:10 mol%): (a) ethylenediamine IL(R_1_, R_2_ = H, H) (IL2, 7, 9, 11–13) mixtures, (b) hydroxyethylenediamine IL(R_1_, R_2_ = H, C_2_H_4_OH) (IL1, 5, 8, 10) mixtures. All measurements were performed at 313 K under dry conditions (water vapor pressure of <0.03 kPa) with a feed gas of CO_2_ 40 Pa in N_2_. The solid line represents the polymer upper boundary reported in Ref. [23]. Closed circles indicate the [C_2_mim][AcO] single system, and a closed triangle indicates the pure diamine IL system.

Next, we examined the effect of spacer length in linear diamine carriers. Figure 3a shows the *P*_CO2_ and *S*_CO2/N2_ for FTMs containing linear diamine ILs with various spacer lengths of cations (*n* = 1–6 in Table 1; IL2, IL7, IL9, and IL11‐IL13) under the dry condition at 313 K. An enlarged view is provided in Figure S1(a). The values of *P*_CO2_ and *S*_CO2/N2_ were shown in Table S1. Compared with the IL2−10 (*n* = 1), increasing spacer lengths markedly improved the separation performance. IL7−10 (*n* = 2) exhibited higher *P*_CO2_ and *S*_CO2/N2_, and IL9−10 (*n* = 3) reached the maximum. Further extension gradually reduced performance (IL11−10, IL12−10, IL13−10). For comparison, the performance of the pure linear diamine IL was also evaluated using IL11 (IL11−100). Both the *P*_CO2_ and *S*_CO2/N2_ of IL11−100 were lower than those of [C_2_mim][AcO] and the IL11‐10 mixed system. Figure 3b presents the results for linear diamine ILs with one hydroxyethyl terminal group on the cation (*n* = 0–3 in Table 1; IL1, IL5, IL8, and IL10). An enlarged view is provided in Figure S1(b). Thus, the alkyl spacer length, along with terminal functional groups, governs CO_2_ separation performance. The optimum spacer length for non‐functional diamine ILs under the present experimental conditions was *n* = 3, whereas for the hydroxyethyl functional ILs, it was *n* = 1 in the present study. Furthermore, the hydroxyethyl diamine IL consistently outperformed their non‐functional counterparts. Hence, the combination of an appropriately extended spacer (*n* = 1) with one hydroxyethyl terminal group provides the best structural configuration for achieving high CO_2_ separation performance.

To clarify the origin of these structural effects, the present results were analyzed within the framework of FTM theory [24, 25, 26, 27]. This theory describes carrier‐mediated transport via the reversible reaction: A (solute) + B (carrier) ⇄ AB (solute–carrier complex) and defines the total permeant flux as the sum of the ordinary solution‐diffusion flux and the facilitated flux. The degree of facilitation was expressed by the facilitation factor *F*, which is the ratio of the total flux to the solution‐diffusion flux. Kemena et al. [26] formulated *F* in terms of three essential dimensionless parameters: *K*, *α*, and *ε*. Here *K* is a dimensionless equilibrium parameter derived from the forward and reverse reaction rate constants and the permeant concentration; *α* quantifies the relative strength of facilitation at the inlet; and *ε* is an inverse Damköhler number incorporating the complex diffusivity, the reverse rate constant, and the membrane thickness. For fixed *ε* and *α*, *F* exhibits a distinct maximum as a function of *K*. In addition, too small *K* leads to incomplete complexation (reaction‐limited), whereas too large values of *K* hinder the reverse step and CO_2_ release (release‐limited). Thus, achieving optimal facilitation requires carrier structures that place the system near the optimal equilibrium parameter, thereby maximizing *F*.

In this study, *α* for IL mixtures containing 10 mol% diamine IL was considered approximately constant. We first investigated the influence of diffusivity. Sharifzadeh et al. [27] demonstrated that datasets with identical *D*_C_/*D*_CO2_ ratios, where *D*_C_ and *D*_CO2_ represent the diffusivities of CO_2_‐carrier complex and free CO_2_, respectively, collapse onto a single master curve of *F* versus *K* when plotted on logarithmic axes. Accordingly, we evaluated *D*_C_/*D*_CO2_ for the IL mixtures studied. Table S2 lists *D*_C_ and *D*_CO2_, calculated using the Stokes–Einstein equation and the Hou‐Baltus correlation [28], respectively, at 313.15 K. As shown in Table S2, *D*_C_/*D*_CO2_ for all mixtures lies in the range 10^−1.6^–10^−1.4^, which can be treated as essentially constant according to the literature [27]. Therefore, the observed differences in *F*—and thus in *P*_CO2_—are attributed primarily to variations in *K*, rather than to changes in the diffusivity related term (*ε*).

**FIGURE 4:**
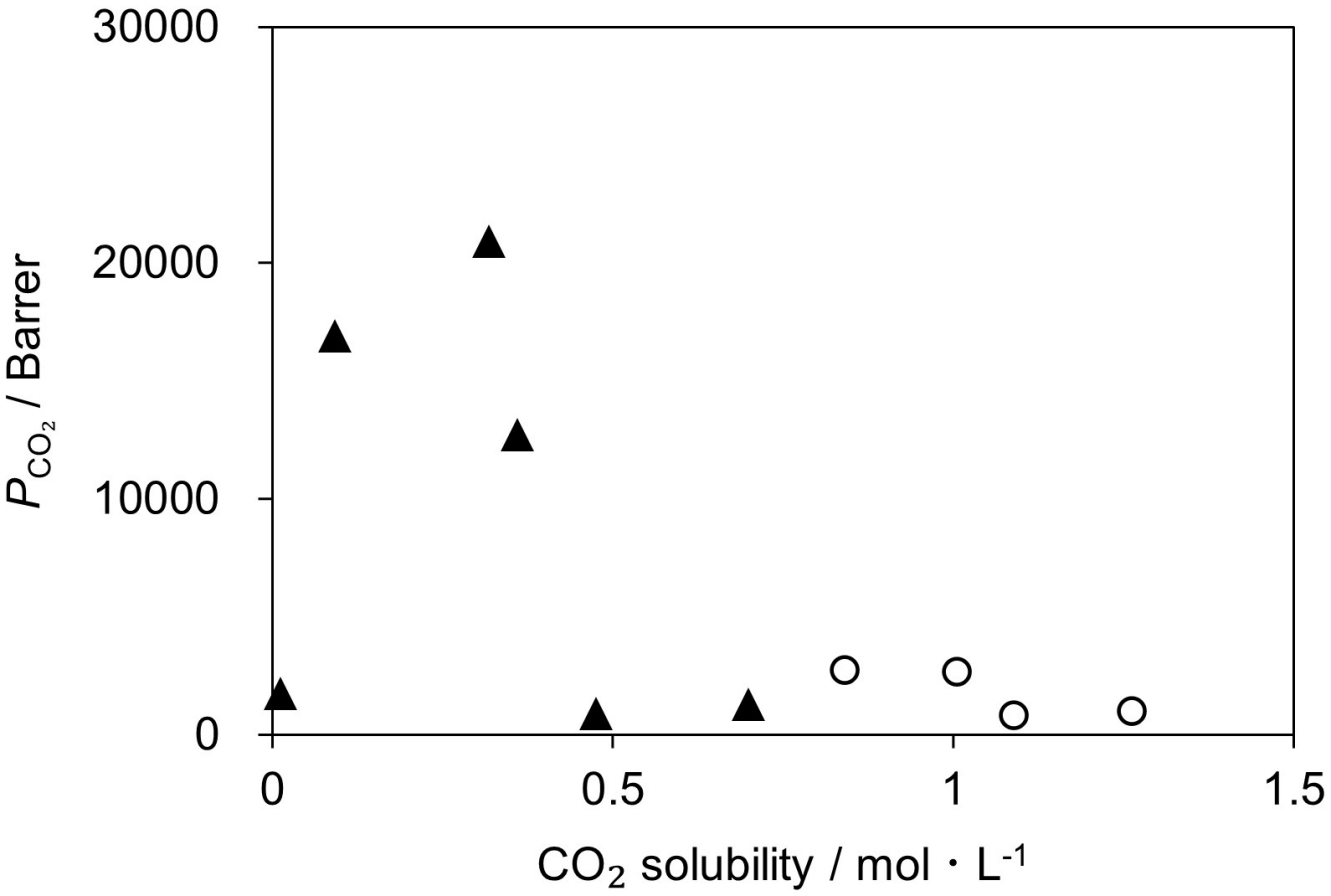
Relationship between *P*_CO2_ and CO_2_ solubility for the [C_2_mim][AcO] single system and [C_2_mim][AcO] + diamine IL mixtures (90:10 mol%); IL2, IL3, IL5, IL6, and IL12. Closed triangles denote data at a CO_2_ partial pressure of 40 Pa, and open circles denote data at 1 kPa.

For subsequent discussion we use the CO_2_ equilibrium solubility, i.e., the permeant concentration, as a proxy for *K*, assuming the forward and reverse rate constants do not vary substantially across the diamine series (same reaction family). Table S3 summarizes CO_2_ solubility of the IL mixtures at CO_2_ pressures of 40 Pa and 1 kPa, obtained from correlations of experimental data [22]. At 40 Pa, CO_2_ solubility decreased in the following order: IL2−10 > IL3−10 > IL12−10 > IL5−10 > IL6−10 > [C_2_mim][AcO], which differs from the order observed for *P*_CO2_. Figure 4 plots *P*_CO2_ as a function of CO_2_ solubility and demonstrates CO_2_ permeability is maximized within an intermediate range of CO_2_ solubility. This behavior indicates higher CO_2_ solubility does not necessarily lead to higher CO_2_ permeability, consistent with the findings of Kemena et al. [26]. A similar trend is presented at 1 kPa, where a distinct maximum appears at a different solubility range, analogous to the case at 40 Pa.

This analysis clarifies the effects of mixing and chemical modifications on CO_2_ permeability. [C_2_mim][AcO], which exhibits the lowest CO_2_ solubility, lies in the reaction‐limited region. In contrast, five IL mixtures (10 mol% diamine IL) absorbed larger amounts of CO_2_ than [C_2_mim][AcO]. IL2−10 and IL3−10, which showed the highest and second‐highest CO_2_ solubility, are likely in release‐limited state, although the ethyl terminal group reduces CO_2_ solubility. For IL12−10, the decyl spacer further lowered solubility compared to the ethyl spacer, resulting in moderately high CO_2_ permeability. The hydroxyethyl group reduced CO_2_ solubility more than the ethyl group and the decyl spacer, yielding a favorable solubility close to the optimum, which leads to the higher CO_2_ permeabilities among the present IL mixtures. This also explains why hydroxyethyl‐functionalized linear diamine ILs exhibited higher CO_2_ permeabilities than their non‐functionalized counterparts. Based on this discussion, the cyclic diamine IL with one hydroxyethyl group (IL15−10) would absorb a favorable amount of CO_2_ compared to the non‐functionalized variant (IL14−10, too high solubility) and two hydroxyethyl functionalized variant (IL16−10, too low solubility).

### 2.2 Temperature Dependence on Membrane Separation Ability

**FIGURE 5:**
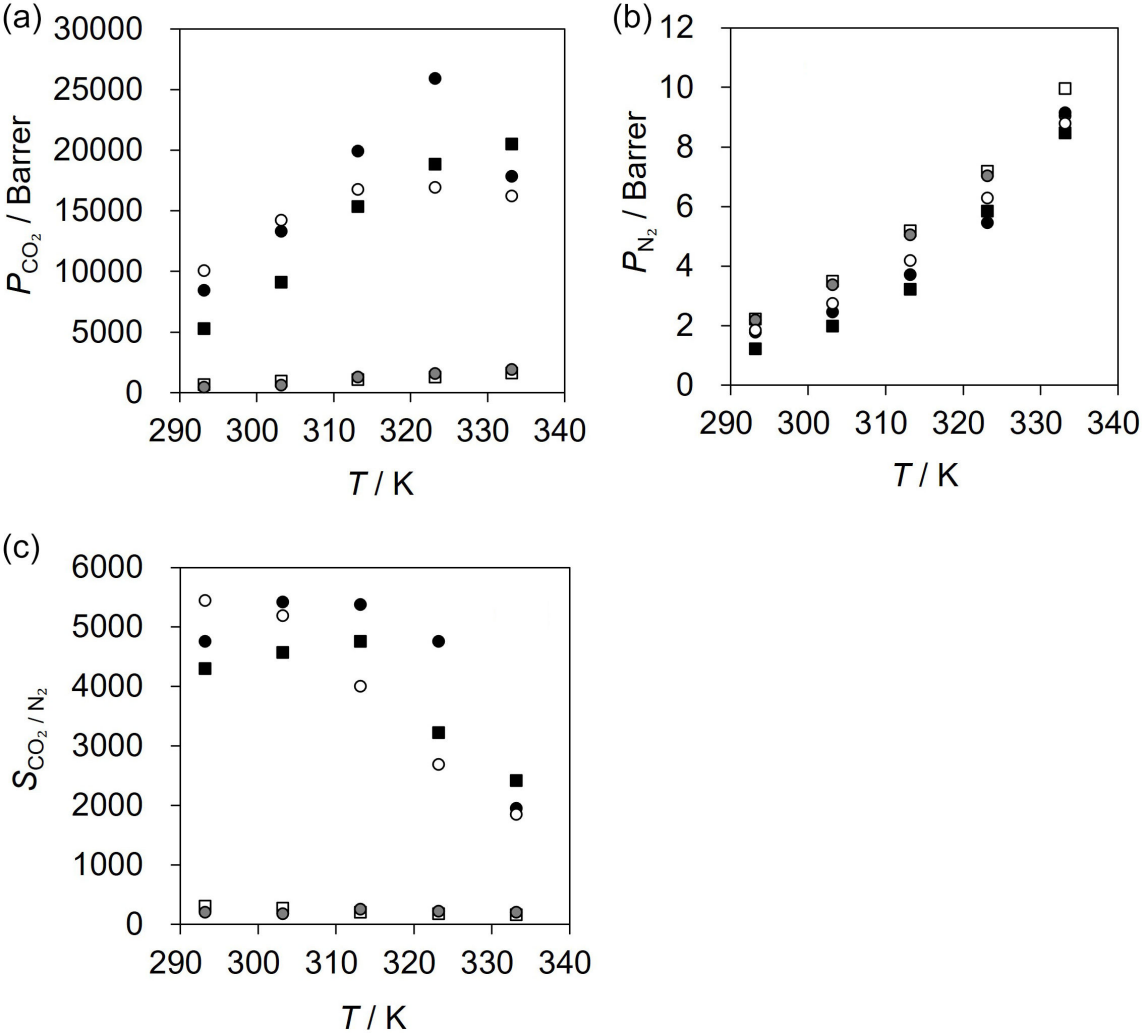
Temperature dependence of separation performance, (a) *P*_CO2_, (b) *P*_N2_, and (c) *S*_CO2/N2_, for the FTMs with [C_2_mim][AcO] + diamine IL mixtures (90:10 mol%), conducted under dry conditions (water vapor pressure of <0.03 kPa) with a feed of CO_2_ (40 Pa in N_2_). Symbols: open square represents IL2−10; closed gray circle represents IL3−10; closed square represents IL12−10; closed circle represents IL5−10; open circle represents IL6−10.

Figure 5a–c show the temperature dependence of CO_2_ and N_2_ permeabilities and CO_2_/ N_2_ selectivity for FTMs containing linear diamine ILs (IL2−10, IL3−10, IL5−10, IL6−10, and IL12−10) measured between 293 K and 333 K. Both CO_2_ and N_2_ permeabilities generally increased with temperature; however, FTMs containing hydroxyethyl‐terminated ILs (IL5−10 and IL6−10) exhibited a decline in *P*_CO2_ above 323 K, indicating strong temperature sensitivity of gas transport in these membranes.

The overall rise in permeability reflects the temperature‐induced reduction in viscosity and density of the IL mixtures, which enhances the diffusivities of both the CO_2_–carrier complex (*D*_C_) and free CO_2_ (*D*_CO2_) by nearly an order of magnitude (Table S4). The temperature dependence of CO_2_ solubility is superimposed on this diffusive acceleration: for chemically reactive amine‐based ILs, CO_2_ solubility decreases with increasing temperature due to the exothermic nature of CO_2_–amine complexation [29]. The net effect depends on the facilitation regime defined by FTM theory—release‐limited versus reaction‐limited—as described in Section 2.1 and the literature [24, 25, 26, 27].

In release‐limited systems, higher temperature reduces excessive CO_2_ solubility toward the optimal range, thereby enhancing decomplexation and increasing *P*_CO2_. In reaction‐limited systems, further loss of CO_2_ solubility moves the system away from optimal complexation despite faster diffusion, causing *P*_CO2_ to plateau or decline. This interplay explains the observed behaviors: IL5−10 shows a pronounced increase in *P*_CO2_ up to 323 K followed by a decrease at 333 K, consistent with a transition from release‐limited to reaction‐limited conditions. IL6−10 remains nearly constant *P*_CO2_ between 313 K and 333 K, indicating compensation between decreasing solubility and increasing diffusivity. IL12−10 exhibits a monotonic increase in *P*_CO2_ across the entire temperature range, consistent with a release‐limited state moving toward the facilitation optimum. IL2−10 and IL3−10 persist in a release‐limited regime over 293–333 K; while *P*_CO2_ increases modestly with temperature, facilitation remains weak and permeability stays far below hydroxyethyl‐substituted systems.

A universal outcome of heating is the monotonic rise of *P*_N2_ unlike *P*_CO2_, yielding a decline in *S*_CO2/N2_ at elevated temperatures. N_2_ transport is governed by solution–diffusion without chemical reaction, so its permeability scales primarily with diffusivity and free volume of IL. In contrast, CO_2_ transport depends on both diffusion and chemical reactions, consequently, selectivity deteriorates when *P*_CO2_ fails to keep pace under unfavorable solubility conditions. From a process design perspective, these findings suggest that CO_2_ permeance and selectivity can be tuned by controlling FTM operating temperature.

### 2.3 Effect of Humidity on Separation Performance

**FIGURE 6:**
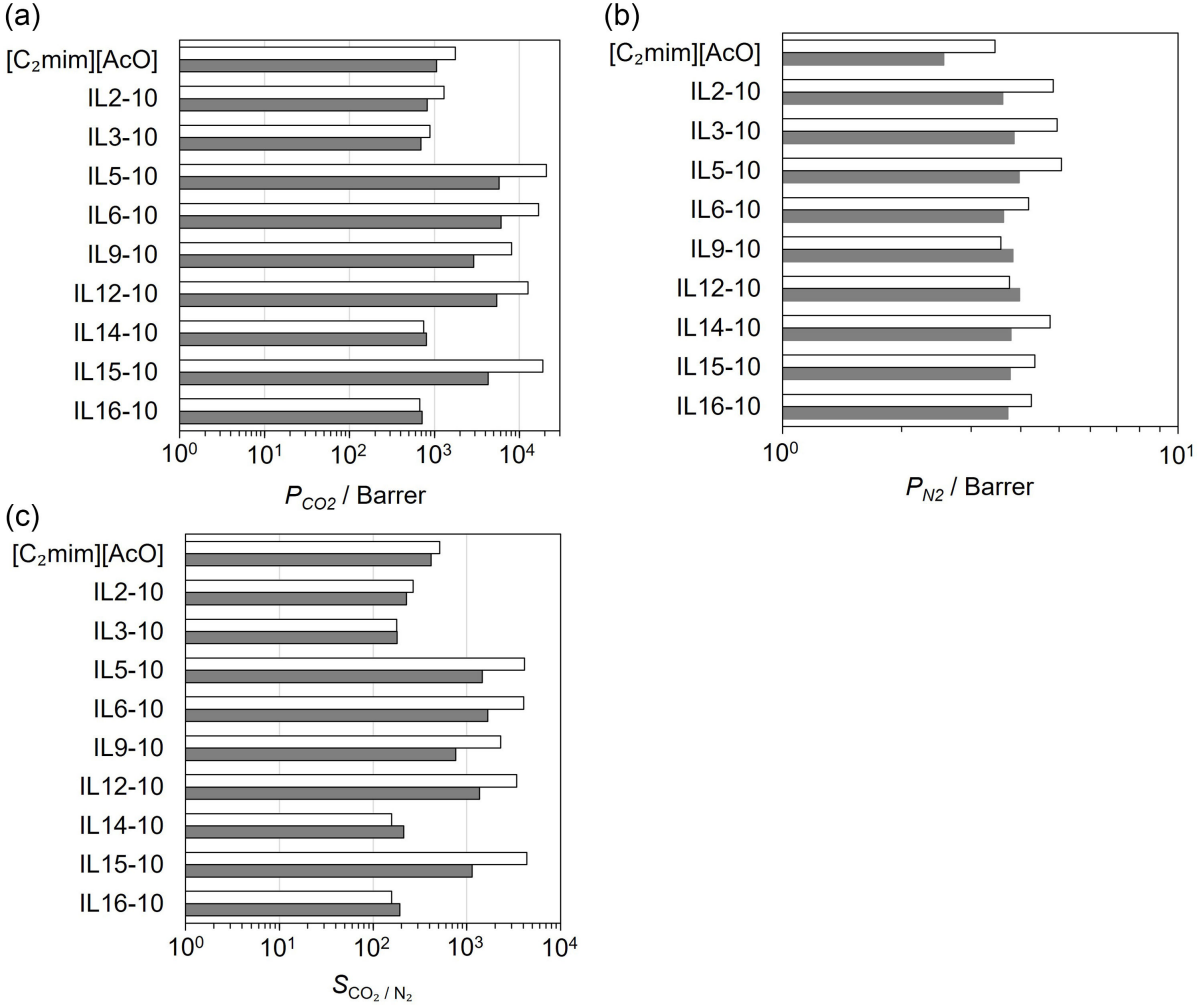
Comparison of separation performance, (a) *P*_CO2_, (b) *P*_N2_, (c) *S*_CO2/N2_, under dry (water vapor pressure <0.03 kPa) and humid (1.8 kPa) conditions for the FTMs with [C_2_mim][AcO] single system and [C_2_mim][AcO] + diamine IL mixtures (90:10 mol%). Measurements were performed at 313 K with a CO_2_ partial pressure of 40 Pa in N_2_. White bars indicate dry conditions, and gray bars indicate humid conditions.

Figure 6a–c show the humidity dependence of CO_2_ and N_2_ permeabilities and CO_2_/N_2_ selectivity for FTMs containing linear diamine ILs (IL2−10, IL3−10, IL5−10, IL6−10, IL9−10, IL12−10) and cyclic diamine ILs (IL14−10, IL15−10, IL16−10) measured at 313 K under dry (<0.03 kPa H_2_O) and humidified (1.8 kPa H_2_O) conditions. Under humidified conditions, both *P*_CO2_ and *S*_CO2/N2_ were smaller than those under dry conditions for nearly all membranes. Significant declines were observed for membranes containing hydroxyethyl‐terminated ILs, except IL16−10, which already exhibited relatively low *P*_CO2_ and *S*_CO2/N2_ under dry conditions. On the other hand, the *P*_N2_ remained low, in the range of 3–4 Barrer, with only minor variations among samples.

**FIGURE 7:**
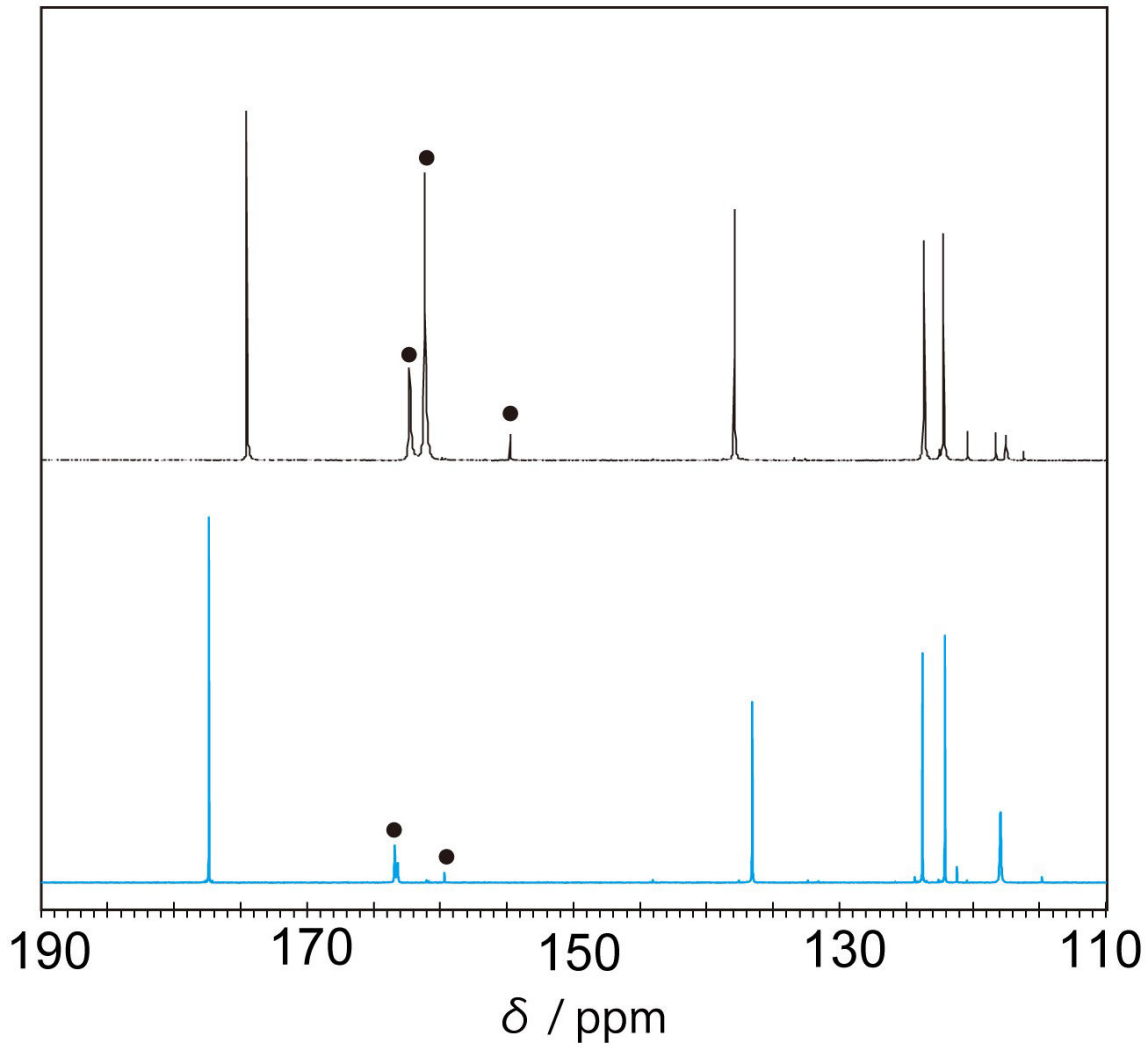
^13^C inverse‐gated decoupled NMR spectra of IL5−10 mixtures after dissolving ^13^CO_2_ under dry (black lines) and humidified (blue lines) conditions. Black dots indicate chemisorbed CO_2_ signals.

As mentioned above, the CO_2_ solubility is one of the key properties related to *P*_CO2_. To clarify the effect of humidity on the CO_2_ solubility, ^13^CO_2_‐saturated mixed IL5−10 systems were analyzed using nuclear magnetic resonance (NMR) spectroscopy. IL5−10 mixture was placed in an NMR tube, and dry or humidified ^13^CO_2_ gas (405 ppm, water vapor partial pressure of 1.8 kPa) was supplied to the mixture. Figure 7 shows the ^13^C inverse‐gated decoupled NMR spectra of the IL5−10 under dry and humidified conditions. Under dry conditions, the chemisorbed products were observed at 162, 161, and 155 ppm, corresponding to the formation of carbamic acid/zwitterionic species and N‐heterocyclic carbene‐CO_2_ adducts [21]. In contrast, under humidified conditions, signals derived from chemisorbed CO_2_ appeared at 163 and 160 ppm. The signal at 163 ppm is consistent with the presence of carbamate species, whereas the peak at 160 ppm is likely attributable to the formation of bicarbonate in the presence of water [30]. The amount of dissolved CO_2_ in the IL mixtures was estimated from the ratio of the peak intensities of chemisorbed CO_2_ and the terminal carbon of the ethyl group in [C_2_mim]^+^. To allow for molar comparison between the CO_2_ and IL species, the NMR integrals of the ^13^CO_2_ peaks were multiplied by 0.011 to account for the natural abundance (1.1%) of the IL signals as shown in our reported study [21]. The resulting value was then normalized by the total weight of IL mixture. The relative amounts of dissolved CO_2_ were estimated to be 0.29 mmol‐CO_2_/g‐total ILs under dry conditions, and 0.04 mmol‐CO_2_/g‐total ILs under humidified conditions, respectively. For IL5−10, the noticeable decrease in CO_2_ solubility in the presence of water is consistent with the lower *P*_CO2_ observed.

The decrease in *P*_CO2_ under humidified conditions can be explained by the reduction in CO_2_ solubility and the *P*_CO2_–CO_2_ solubility relationship discussed in Figure 4. For the IL5−10 system, increasing humidity lowers the CO_2_ solubility from the optimum range for facilitated transport, which is consistent with a shift from a release‐limited regime toward a reaction‐limited regime. This interpretation aligns with the observed decrease in both *P*_CO2_ and *S*_CO2/N2_ for IL5−10 under humidified conditions.

To improve *P*_CO2_ under humidified conditions, we varied the molar fraction of the mobile carrier (IL5) to optimize CO_2_ solubility in the IL phase. NMR analysis estimated the dissolved CO_2_ amounts in IL5−30 to be 0.16 mmol‐CO_2_ g^−1^‐IL under dry conditions and 0.10 mmol‐CO_2_ g^−1^‐IL under humidified conditions, whereas for IL5−70 the corresponding values were 0.02 and 0.01 mmol‐CO_2_ g^−1^‐IL, respectively. Thus, under dry conditions, IL5−10 exhibited the highest CO_2_ solubility among these compositions, whereas under humidified conditions, IL5−30 exhibited the highest CO_2_ solubility.

**FIGURE 8:**
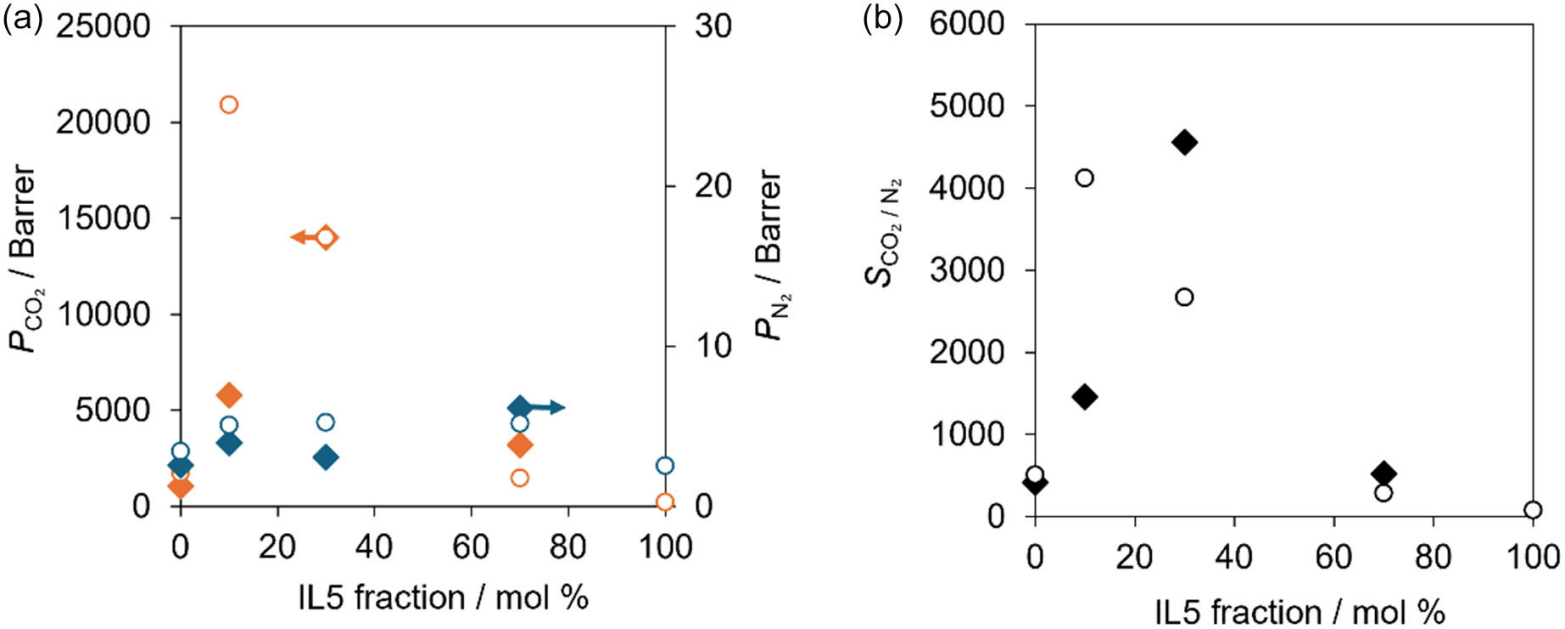
Separation performance of the FTMs with [C_2_mim][AcO] + IL5 at different molar fraction of IL5: (a) *P*_CO2_ (orange symbols) and *P*_N2_ (blue symbols); (b) *S*_CO2/N2_. Measurements were conducted at 313 K with a CO_2_ partial pressure of 40 Pa in N_2_. Closed diamonds indicate humid conditions (water vapor pressure 1.8 kPa); open circles indicate dry conditions (<0.03 kPa) [21].

Figure 8 shows the *P*_CO2_, *P*_N2_, and *S*_CO2/N2_ of the IL5‐based FTMs as a function of the molar fraction of IL5. Under dry conditions, IL5−10 exhibited the highest *P*_CO2_ and *S*_CO2/N2_, whereas further increases in the IL5 fraction caused a gradual decrease in *P*_CO2_. Under humidified conditions, IL5−10 showed a pronounced decrease in both *P*_CO2_ and *S*_CO2/N2_. In contrast, for IL5−30, *P*_CO2_ was retained under both dry and humidified conditions, and *S*_CO2/N2_ increased under humidified conditions because *P*_N2_ decreased while *P*_CO2_ was maintained. Although the CO_2_ solubility of IL5−30 decreased under humidified conditions relative to that under dry conditions, *P*_CO2_ remained comparable, suggesting that the CO_2_ solubility still lies within the high‐*P*_CO2_ range shown in Figure 4. This behavior also implies that additional factors, such as water‐induced enhancement of diffusion via viscosity reduction, contribute to the observed *P*_CO2_. A further increase in the IL5 fraction to 70 mol% (IL5−70) reduced both *P*_CO2_ and *S*_CO2/N2_ under humidified conditions. Nevertheless, both values under humidified conditions exceeded those obtained under dry conditions, likely reflecting the sensitivity of gas diffusivity of the IL phase to water uptake under this highly IL5‐rich composition.

## 3 Conclusion

In this study, FTMs composed of mixtures of diamine‐functionalized ILs and [C_2_mim][AcO] were evaluated for CO_2_ separation under low CO_2_ partial pressures relevant to DAC. We systematically examined the effects of the diamine carrier structure on the gas separation performance by varying the terminal functional groups (hydrogen, ethyl, hydroxyethyl), main framework (linear or cyclic diamine cation), and, for the linear series, the spacer length between the amino groups. FTMs incorporating diamine carriers bearing one hydroxyethyl terminal group exhibited markedly higher *P*_CO2_ and *S*_CO2/N2_ than systems with ethyl‐ or hydrogen‐terminated analogs. Extending the spacer length in linear diamine carriers further improved the performance and yielded optimal ranges at intermediate chain lengths. Although *P*_CO2_ and *S*_CO2/N2_ generally increased with temperature, the membranes containing hydroxyethyl‐terminated carriers exhibited a decline at higher temperatures. These trends are consistent with the FTM framework in which CO_2_ transport depends on the balance between a release‐limited regime at high CO_2_ solubility and a reaction‐limited regime at low CO_2_ solubility, with maximum *P*_CO2_ obtained in an optimum solubility range. When CO_2_ solubility is excessively high, *P*_CO2_ remains relatively low; by moderating the solubility through molecular modification by introducing hydroxyethyl group(s) or adjusting the diamine spacer length, the system enters an optimal range that affords higher *P*_CO2_. Conversely, when CO_2_ solubility falls below the optimum range, the system shifts to a reaction‐limited regime and *P*_CO2_ decreases. Under humidified conditions, for membranes containing 10 mol% diamine‐functionalized IL, both *P*_CO2_ and *S*_CO2/N2_ decreased owing to the reduced CO_2_ solubility. Nevertheless, by increasing the diamine‐functionalized IL fraction to 30 mol%, the performance losses were mitigated. Notably, compositions enriched in the diamine carrier preserved *P*_CO2_ while providing higher *S*_CO2/N2_ under humidified conditions compared with the dry state, indicating that the separation performance is adjustable according to the operating humidity.

## 4 Experimental

### 4.1 Synthesis of the ILs

The ILs used in this study are shown in Figure 1 and Table 1. The imidazolium‐based IL, 1‐ethyl‐3‐methylimidazolium acetate ([C_2_mim][AcO]), was purchased from IoLiTec (Germany) and used without further purification. All the diamine‐functionalized ILs contained bis(trifluoromethanesulfonyl)imide ([Tf_2_N]^‐^), and their corresponding cations are illustrated as [X]^+^ in Figure 1. Diamine‐functionalized ILs were synthesized by mixing equimolar amounts of various diamines with a methanolic solution of H[Tf_2_N], followed by stirring at room temperature for more than 12 h. The solvent was then removed under reduced pressure to obtain crude ILs. After vacuum drying of each IL, mixed IL systems were prepared by blending diamine‐functionalized ILs with [C_2_mim][AcO] under a nitrogen atmosphere (dew point <−30°C).

The synthesized ILs were characterized by NMR spectroscopy (JEOL, ECA‐600, Japan and Bruker, Avance 400, Germany) and elemental analysis (Elementar, vario MICRO cube, Germany) to confirm their purities. The water content was measured using Karl Fischer titration (Kyoto Electronics, MKC‐510, Japan). Details are provided in the Supporting Information.

### 4.2 Measurement of CO_2_/N_2_ Gas Permeability for FTMs

A schematic diagram of the CO_2_/N_2_ mixed gas permeation measurement setup is shown in Figure S2. FTMs were prepared as supported IL membrane (SILM), following the procedure described in our previous report [21]. The IL was impregnated into a porous PTFE membrane support, achieving a filling rate of 98%–99%. The filling rate was defined as the volume fraction of the IL loaded into the void space of the PTFE filter. The thickness of the resulting SILMs was 30 μm, and no change in the membrane thickness was observed before and after IL impregnation.

The membrane was mounted in a stainless‐steel permeation cell and placed either in a temperature‐controlled water bath equipped with a circulator (AS ONE, MCX‐250, Japan) at 293–303 K or in a thermostatic oven (ISUZU, DSN‐113, Japan) maintained at temperatures of 313–333 K. Mixed‐gas permeability tests were conducted using a CO_2_/N_2_ mixture, with the feed‐side flow rate set to 400 cm^3^(STP)/min. The permeate side was swept with He as the sweep gas. The sweep gas flow rate was adjusted in the range of 20–100 cm^3^(STP)/min to match the permeation flux and ensure accurate quantification. The feed gas composition was controlled using mass flow controllers (MFC, HORIBA STEC, SEC‐E40, Japan), and both the feed and sweep side pressures were maintained close to atmospheric pressure. The temperature, humidity, and dew point inside the gas line were measured using humidity‐temperature probes (Rotronic, HC2‐IE102‐M, Switzerland). The outlet gas flow rates were measured using a film flow meter (HORIBA STEC, SF‐1U, Japan). The compositions of the feed and permeate gases (CO_2_ and N_2_) were analyzed by gas chromatography (GC, GC‐8A, Shimadzu, Japan) using a Shincarbon ST column (Shinwa Chemical Industries, Japan). Each GC analysis was repeated at least three times, and the data were accepted only when the variation between the sequential peak areas of CO_2_ and N_2_ was less than 1%. All permeability and selectivity values were determined from three independent permeation measurements. The overall experimental uncertainty was ±2% for both CO_2_ permeability and CO_2_/N_2_ selectivity.

Permeation experiments under humidified conditions were also carried out to evaluate the membrane performance in environments simulating atmospheric moisture. To introduce water vapor into the feed gas, a vaporizer was used by wrapping the feed‐gas line with a heating mantle and maintaining it at 393 K to ensure complete vaporization of water. Water was injected into the vaporizer using a syringe pump (YMC, YSP‐101, USA), enabling controlled and continuous humidification into the gas stream. The relative humidity of the gas mixture was continuously monitored using a CO_2_/H_2_O sensor (LICOR, LI‐850, USA) to ensure consistency. The remaining set‐up and operating conditions were identical to those used in the dry gas experiments.

### 4.3 Calculation of Diffusion Coefficients

The diffusion coefficients of the CO_2_–carrier complexes were calculated using the Stokes–Einstein equation. The viscosity values were taken from our previous study [22], where they were measured for the neat ILs without CO_2_ loading. This is justified because our previous study confirmed that, under the conditions of this study, CO_2_ absorption did not alter the bulk viscosity of the IL phase [21].

To compute the hydrodynamic radii used in the Stokes–Einstein equation, van der Waals volumes (*V*_IL_) of the carrier molecules were obtained using Gaussian 16 (Revision 1.1) with geometry optimization at the ωB97X‐D/6‐311++G(d, p) level. Molecular volumes were determined from the 0.001 e·bohr^−3^ isodensity surface and then converted to spherical‐equivalent volumes to obtain the hydrodynamic radius, *r*_ion_, assuming the carrier molecule behaves as a sphere in the liquid phase (Table S5).

This hydrodynamic radius *r*_ion_, together with the viscosity *η* and absolute temperature *T*, was substituted into the Stokes–Einstein equation to estimate the diffusion coefficient *D*. These calculated diffusion coefficients were used for the analysis of facilitated transport in Section 2.1.

### 4.4 NMR Analysis of the CO_2_ Saturated IL Mixtures Under Dry and Humidified Conditions

A certain amount of the IL mixture was placed in an NMR tube with a sealed capillary containing acetonitrile‐*d*_3_. The tube was then inserted into a temperature‐controlled chamber (ESPEC CORP., SU‐222, Japan) maintained at 313.2 K. The standard ^13^CO_2_ gas (405 ppm) was humidified by passing it through two glass bubblers filled with Milli‐Q water. The partial pressure of water vapor was adjusted to 1.8 kPa, as confirmed using a humidity sensor (Vaisala, HMP3, Finland). The humidified ^13^CO_2_ gas was slowly bubbled into the IL sample through a stainless‐steel needle for 1 week until saturation was achieved. The saturated solution was then analyzed by ^13^C NMR spectroscopy using an inverse‐gated decoupling pulse sequence.

## Supporting Information

Additional supporting information can be found online in the Supporting Information section.

## Supporting information

Supplementary Material
