# Supplementary material for: Mobile Carrier Structure for Facilitated CO2 Transport in Mixed Ionic Liquid Membrane Composed of 1‐Ethyl‐3‐Methylimidazolium Acetate and Diamine‐Functionalized Ionic Liquids
Source: ChemSusChem. 2026 May 15;19(10):e70685. doi: 10.1002/cssc.70685 (PMC13177167; doi:10.1002/cssc.70685)
Supplement: Supplementary file 1 — Supplementary Material [file CSSC-19-e70685-s001.pdf]

Supporting Information

for

Mobile carrier structure for facilitated CO_2 transport in mixed ionic liquid membrane composed of 1-ethyl-3-methylimidazolium acetate and diamine-functionalized ionic liquids

Yu Nagai Kanasaki[a], Yuki Kohno*[a], and Takashi Makino*[a]

[a]National Institute of Advanced Industrial Science and Technology (AIST),

4-2-1, Nigatake, Miyagino-Ku, Sendai 983-8551, Japan.

* corresponding author

Materials

Ethylenediamine anhydrous (>98.0%), *N*-(2-hydroxyethyl)ethylenediamine (>99.0%), *N*,*N*'-bis(2-hydroxyethyl)ethylenediamine (>98.0%), *N*-ethylethylenediamine (>99.0%), *N*,*N*'-diethylethylenediamine (>95.0%), 1,4-butanediamine (>98.0%), 1,6-hexanediamine (>99.0%), 1,8-octanediamine (>98.0%), 1,10-decanediamine (>98.0%), 1,12-dodecanediamine (>98.0%), piperazine anhydrous (>98.0%), 1-(2-hydroxyethyl)piperazine (>99.0%), and 1,4-bis(2-hydroxyethyl)piperazine (>98.0%) were purchased from Tokyo Chemical Industry Co., Ltd., Japan. 2-Hydroxyethylhydrazine (>95%) was purchased from Merck KGaA, Germany. Bis(trifluoromethanesulfonyl)imide (H[Tf_2N], >98.0%) and 2-chloroethanol (95%) were purchased from Kanto Chemical Co., Inc., Japan. 1-Ethyl-3-methylimidazolium acetate ([C_2mim][AcO], >99.6%) was purchased from IoLiTec GmbH, Germany. CO_2 (>99.995%) was supplied by Nippon Ekitan Corp., Japan. He (>99.99995%) was supplied by Taiyo Nippon Sanso Corp., Japan. $^{13}CO_2/N_2$ (405 ppm) standard gas was supplied by Sumitomo Seika Chemicals Co., Ltd., Japan.

Synthesis of the ILs

Aminium ILs used in this study were prepared by the neutralization of diamines with equimolar amounts of H[Tf_2N]. For example, in the preparation of IL5, *N*-(2-hydroxyethyl)ethylenediamine and H[Tf_2N] were mixed in ethanol at a molar ratio of 1.0/1.0 while cooling the solution in an ice bath, and subsequently, the solution was stirred for >2 h. After this time, the solvent was evaporated with a rotary evaporator, and the sample was dried in vacuo to obtain IL5. For the synthesis of IL8 or IL10, *N*-(2-aminobutyl)ethanolamine or *N*-(6-aminohexyl)ethanolamine was prepared by reacting 1,4-butanediamine or 1,6-hexanediamine with 2-chloroethanol in ethanol with a molar ratio of 1/0.3, followed by distillation to obtain the amine precursors. The subsequent procedure was the same as that used for IL5.

Characterization of ILs

2-(2-hydroxyethyl)hydrazin-1-ium bis(trifluoromethanesulfonyl)imide (IL1). ^{1}H

NMR (400 MHz, D_2O): δ = 3.20 (t, J = 5.0 Hz, 2H), 3.80 (t, J = 5.0 Hz, 2H); ^{13}C NMR (100 MHz, D_2O): δ = 52.27, 56.23, 119.24 (q, J = 317.7 Hz). Found: C, 12.90; H, 2.55; N, 11.82. Calc. for $C_4H_9F_6N_3O_5S_2$: C, 13.45; H, 2.54; N, 11.76%. The water content is not reported because Karl Fischer titration failed due to precipitation of the samples in the cell.

2-Aminoethan-1-aminium bis(trifluoromethanesulfonyl)imide (IL2). ^{1}H NMR (400 MHz, D_2O): δ = 2.98 (br, 4H); ^{13}C NMR (100 MHz, D_2O): δ = 39.75, 119.31 (q, J = 317.7 Hz). Found: C, 13.65; H, 2.74; N, 12.16. Calc. for $C_4H_9F_6N_3O_4S_2$: C, 14.08; H, 2.66; N, 12.31%. Water content: 12 ppm.

2-(Ethylamino)ethan-1-aminium bis(trifluoromethanesulfonyl)imide (IL3). ^{1}H NMR (400 MHz, D_2O): δ = 1.39 (t, J = 7.2 Hz, 3H), 3.14 (m, 6H); ^{13}C NMR (100 MHz, D_2O): δ = 11.19, 37.51, 43.13, 48.28, 119.54 (q, J = 317.7 Hz). Found: C, 19.59; H, 3.98; N, 11.19. Calc. for $C_6H_{13}F_6N_3O_4S_2$: C, 19.51; H, 3.55; N, 11.38%. Water content: 28 ppm.

***N*-Ethyl-2-(ethylamino)ethan-1-aminium bis(trifluoromethanesulfonyl)imide (IL4).** ^{1}H NMR (400 MHz, neat, C_6D_6 as external standard): δ = 1.11 (t, J = 7.2, 6H), 2.76 (q, J = 7.1 Hz, 4H), 2.84 (br, 4H); ^{13}C NMR (100 MHz, neat, C_6D_6 as external standard): δ = 12.24, 42.91, 45.00, 119.74 (q, J = 318.3 Hz). Found: C, 23.68; H, 4.68; N, 10.77. Calc. for $C_8H_{17}F_6N_3O_4S_2$: C, 24.18; H, 4.31; N, 10.57%. Water content: 35 ppm.

2-[(2-Hydroxyethyl)amino]ethan-1-aminium bis(trifluoromethanesulfonyl)imide (IL5). ^{1}H NMR (400 MHz, D_2O): δ = 2.89 (t, J = 4.6 Hz, 2H), 2.98 (t, J = 6.4 Hz, 2H), 3.08 (t, J = 6.4 Hz, 2H), 3.73 (t, J = 4.8 Hz, 2H); ^{13}C NMR (100 MHz, D_2O): δ = 38.19, 46.42, 49.64, 59.22, 119.36 (q, J = 317.7 Hz). Found: C, 16.81; H, 3.82; N, 10.53. Calc. for $C_6H_{13}F_6N_3O_5S_2\cdot 2H_2O$: C, 17.10; H, 4.07; N, 9.97%. Water content: 15 ppm.

2-Hydroxy-*N*-[2-[(2-hydroxyethyl)amino]ethyl]ethan-1-aminium bis(trifluoromethanesulfonyl)imide (IL6). ^{1}H NMR (400 MHz, D_2O): δ = 3.11 (br, 4H), 3.20 (br, 4H), 3.89 (br, 4H); ^{13}C NMR (100 MHz, D_2O): δ = 45.40, 49.65, 58.62, 119.51 (q, J = 318.0 Hz). Found: C, 20.22; H, 4.51; N, 9.34. Calc. for $C_8H_{17}F_6N_3O_6S_2\cdot 2H_2O$: C, 20.65; H, 4.55; N, 9.03%. Water content: 29 ppm.

4-Aminobutan-1-aminium bis(trifluoromethanesulfonyl)imide (IL7). ^{1}H NMR (400 MHz, D_2O): δ = 1.55 (br, 2H), 2.77 (br, 2H); ^{13}C NMR (100 MHz, D_2O): δ = 26.43, 39.61,

119.27 (q, J = 317.7 Hz). Found: C, 19.07; H, 3.69; N, 11.67. Calc. for $C_6H_{13}F_6N_3O_4S_2$: C, 19.51; H, 3.55; N, 11.38%. Water content: 15 ppm.

4-[(2-Hydroxyethyl)amino]butan-1-aminium bis(trifluoromethanesulfonyl)imide (IL8). ^{1}H NMR (400 MHz, D_2O): δ = 1.64 (br, 4H), 2.81 (m, 6H), 3.73 (br, 2H); ^{13}C NMR (100 MHz, D_2O): δ = 24.84, 25.61, 39.44, 47.53, 49.57, 59.04, 119.30 (q, J = 317.7 Hz). Found: C, 22.76; H, 4.28; N, 10.17. Calc. for $C_8H_{17}F_6N_3O_5S_2$: C, 23.25; H, 4.15; N, 10.17%. Water content: 30 ppm.

6-Aminohexan-1-aminium bis(trifluoromethanesulfonyl)imide (IL9). ^{1}H NMR (400 MHz, D_2O): δ = 1.47 (br, 4H), 1.66 (t, J = 6.0 Hz, 4H), 2.90 (t, J = 7.4 Hz, 4H); ^{13}C NMR (100 MHz, D_2O): δ = 25.60, 29.21, 40.12, 119.44 (q, J = 317.7 Hz). Found: C, 25.17; H, 4.56; N, 10.79. Calc. for $C_8H_{17}F_6N_3O_4S_2$: C, 24.18; H, 4.31; N, 10.58%. Water content: 46 ppm.

6-[(2-Hydroxyethyl)amino]hexan-1-aminium bis(trifluoromethanesulfonyl)imide (IL10). ^{1}H NMR (400 MHz, D_2O): δ = 1.36 (br, 4H), 1.57 (t, J = 7.2 Hz, 4H), 2.71 (t, J = 7.2 Hz, 2H), 2.85 (t, J = 7.6 Hz, 4H), 3.71 (br, 2H); ^{13}C NMR (100 MHz, D_2O): δ = 25.50, 25.83, 27.43, 28.17, 39.81, 48.07, 49.67, 59.18, 119.35 (q, J = 317.7 Hz). Found: C, 26.95; H, 4.70; N, 9.77. Calc. for $C_{10}H_{21}F_6N_3O_5S_2$: C, 27.21; H, 4.80; N, 9.52%. Water content: 35 ppm.

8-Aminooctan-1-aminium bis(trifluoromethanesulfonyl)imide (IL11). ^{1}H NMR (400 MHz, MeOD): δ = 1.39 (br, 8H), 1.58 (t, J = 6.0 Hz, 4H), 2.78 (t, J = 7.4 Hz, 4H); ^{13}C NMR (100 MHz, MeOD): δ = 26.20, 28.81, 29.80, 40.28, 119.81 (q, J = 318.3 Hz). Found: C, 28.00; H, 4.83; N, 10.22. Calc. for $C_{10}H_{21}F_6N_3O_4S_2$: C, 28.23; H, 4.98; N, 9.88%. Water content: 34 ppm.

10-Aminodecan-1-aminium bis(trifluoromethanesulfonyl)imide (IL12). ^{1}H NMR (400 MHz, $CDCl_3$): δ = 1.27 (br, 12H), 1.49 (br, 4H), 2.71 (br 4H); ^{13}C NMR (100 MHz, $CDCl_3$): δ = 26.31, 28.85, 29.02, 30.36, 40.36, 119.66 (q, J = 319.0 Hz). Found: C, 32.04; H, 5.51; N, 9.56. Calc. for $C_{12}H_{25}F_6N_3O_4S_2$: C, 31.78; H, 5.56; N, 9.27%. Water content: 32 ppm.

12-Aminododecan-1-aminium bis(trifluoromethanesulfonyl)imide (IL13). ^{1}H NMR (400 MHz, MeOD): δ = 1.34 (br, 16H), 1.56 (br, 4H), 2.77 (t, J = 7.4 Hz, 4H); ^{13}C NMR

(100 MHz, MeOD): δ = 26.34, 29.02, 29.19, 29.25, 29.93, 40.34, 119.81 (q, J = 318.7 Hz). Found: C, 34.69; H, 5.85; N, 9.25. Calc. for $C_{14}H_{29}F_6N_3O_4S_2$: C, 34.92; H, 6.07; N, 8.73%. The water content is not reported because Karl Fischer titration failed due to precipitation of the samples in the cell.

Piperazin-1-ium bis(trifluoromethanesulfonyl)imide (IL14). ^{1}H NMR (400 MHz, D_2O): δ = 3.22 (br, 8H); ^{13}C NMR (100 MHz, D_2O): δ = 42.79, 119.44 (q, J = 318.0 Hz). Found: C, 19.77; H, 3.14; N, 11.53. Calc. for $C_6H_{11}F_6N_3O_4S_2$: C, 19.62; H, 3.02; N, 11.44%. Water content: 26 ppm.

4-(2-hydroxyethyl)piperazin-1-ium bis(trifluoromethanesulfonyl)imide (IL15). ^{1}H NMR (400 MHz, D_2O): δ = 2.82 (t, J = 5.8 Hz, 2H), 2.96 (br, 4H), 3.36 (t, J = 4.6 Hz, 4H), 3.86 (t, J = 5.6 Hz, 2H); ^{13}C NMR (100 MHz, D_2O): δ = 43.13, 49.76, 57.84, 58.75, 119.43 (q, J = 318.0 Hz). Found: C, 24.25; H, 3.99; N, 10.44. Calc. for $C_8H_{15}F_6N_3O_5S_2$: C, 23.36; H, 3.67; N, 10.22%. Water content: 19 ppm.

1,4-Bis(2-hydroxyethyl)piperazin-1-ium bis(trifluoromethanesulfonyl)imide (IL16). ^{1}H NMR (400 MHz, D_2O): δ = 3.18 (br, 12H), 3.94 (br, 4H); ^{13}C NMR (100 MHz, D_2O): δ=50.56, 56.64, 58.17, 119.44 (q, J = 318.0 Hz). Found: C, 27.12; H, 4.62; N, 9.30. Calc. for $C_{10}H_{19}F_6N_3O_6S_2$: C, 26.38; H, 4.21; N, 9.23%. Water content: 43 ppm.

Table S1. Gas permeability and selectivity of the FTMs with [C_2mim][AcO] + diamine IL mixtures (90:10 mol %) at 313.15 K under dry conditions (water vapor <0.03 kPa) with a feed gas of CO_2 40 Pa in N_2.

IL	P_{CO_2} / Barrer	S_{CO_2/N_2} -
[C_2mim][AcO]	1760	511
IL1-10	1463	281
IL2-10	1299	268
IL3-10	888	179
IL4-10	1117	256
IL5-10	20902	4119
IL5-100	206[a]	82[a]
IL6-10	16895	4035
IL7-10	6247	1289
IL8-10	16959	4011
IL9-10	13249	3212
IL10-10	17963	3912
IL11-10	12887	3517
IL11-100	472	111
IL12-10	12717	3392
IL13-10	11810	2951
IL14-10	749	158
IL15-10	18917	4350
IL16-10	673	158

[a]Data taken from Ref. [21].

Table S2. Diffusion coefficients of CO_2 carrier in the IL mixtures, D_C, calculated using the Stokes–Einstein relation, the CO_2 diffusivities D_{CO_2} estimated from the Hou–Baltus correlation, and the corresponding ratios D_C/D_{CO_2} at 313.15 K.

IL	Diffusion coefficient of CO_2 carrier, D_C /10^{-11} m^2 s^{-1}	Diffusion coefficient of CO_2 in ILs, D_{CO_2} /10^{-11} m^2 s^{-1}	D_C/D_{CO_2}
[C_2mim][AcO]	1.66	38.8	$10^{-1.4}$
IL2-10	1.43	44.5	$10^{-1.5}$
IL3-10	1.34	44.6	$10^{-1.5}$
IL5-10	1.10	41.4	$10^{-1.6}$
IL6-10	1.18	44.6	$10^{-1.6}$
IL12-10	0.75	31.6	$10^{-1.6}$

Table S3. CO_2 solubility for the [C_2mim][AcO] single system and [C_2mim][AcO] + diamine IL mixtures (90:10 mol%) at a CO_2 partial pressure of 40 Pa and 1 kPa at 313.2 K under dry conditions.

IL	CO_2 solubility at 40Pa CO_2 / mol L^{-1}	CO_2 solubility at 1 kPa CO_2 / mol L^{-1}
[C_2mim][AcO]	0.012	-
IL2-10	0.70	1.0
IL3-10	0.48	1.3
IL5-10	0.32	1.1
IL6-10	0.092	0.84
IL12-10	0.36	-

Table S4. Temperature dependence of diffusion coefficient of CO_2 carrier, D_c

Temperature /K	Diffusion coefficient of CO_2 carrier, D_c /10^{-11} m^2 s^{-1}				
	IL2-10	IL3-10	IL5-10	IL6-10	IL12-10
293.15	0.34	0.34	0.26	0.32	-
303.15	0.72	0.68	0.54	0.63	-
313.15	1.43	1.34	1.10	1.18	0.75
323.15	2.14	2.02	1.67	1.80	1.26
333.15	3.33	3.16	2.62	2.73	1.99

Table S5. Properties for calculation of diffusion coefficients of the mixed ILs calculated using the Stokes–Einstein relation at 313.15 K.

IL	Viscosity, η / mPa s	Van der Waals volumes, V_{RTIL} / cm^3 mol^{-1}	Hydrodynamic radius, r_{ion} / m
$[C_2mim][AcO]$	48.3	101.8	0.343
IL2-10	69.4	53.6	0.277
IL3-10	63.7	85.3	0.323
IL5-10	74.9	93.3	0.333
IL6-10	63.9	121.4	0.364
IL12-10	90.0	170.7	0.408

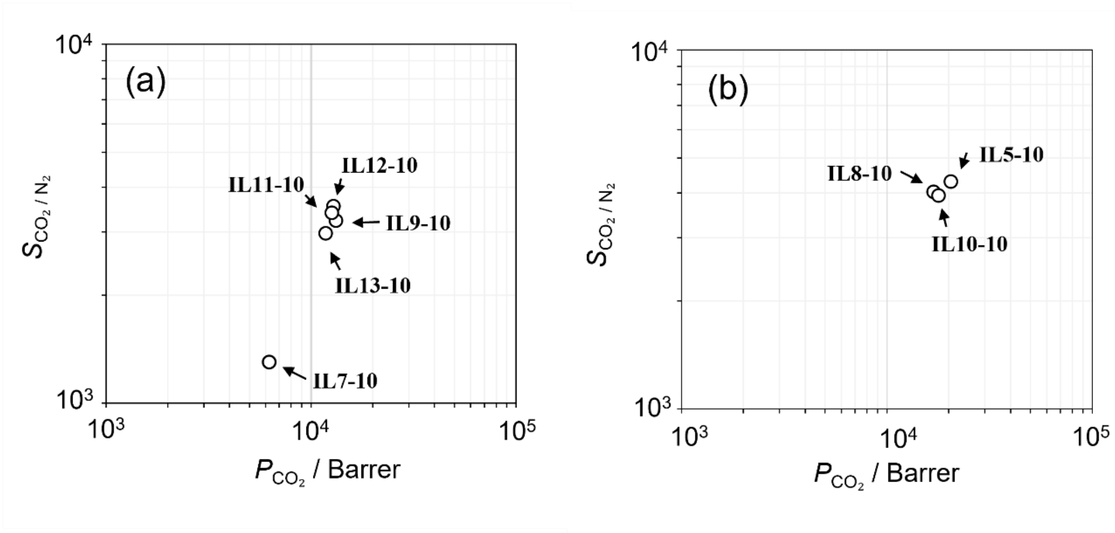

Figure S1. Enlarged view of Figure 3. Robeson plots of the FTMs with [C_2mim][AcO] + diamine IL mixtures (90:10 mol %): (a) ethylenediamine ILs (IL2, 7, 9, 11–13) mixtures, (b) hydroxyethylenediamine ILs (IL1, 5, 8, 10) mixtures. All measurements were performed at 313 K under dry conditions with a feed gas of CO_2 40 Pa in N_2.

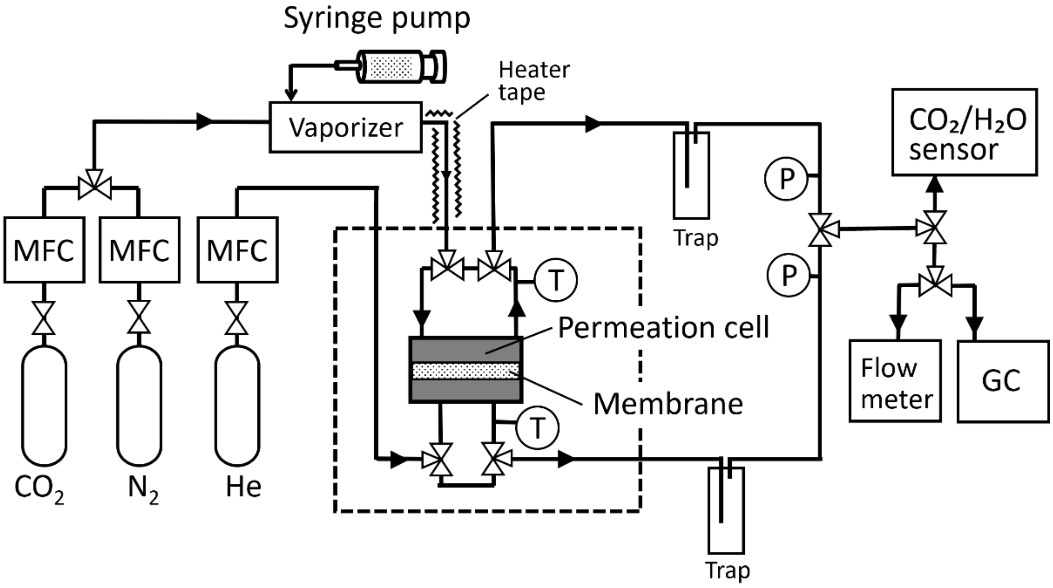

Figure S2. Schematic image of the gas separation evaluation set-up.
